# Supplementary material for: ImmunoSep (Personalised Immunotherapy in Sepsis) international double-blind, double-dummy, placebo-controlled randomised clinical trial: study protocol
Source: BMJ Open. 2022 Dec 20;12(12):e067251. doi: 10.1136/bmjopen-2022-067251 (PMC9772655; doi:10.1136/bmjopen-2022-067251)
Supplement: Author's manuscript [file bmjopen-2022-067251.draft_revisions.pdf]

BMJ Open

BMJ Open is committed to open peer review. As part of this commitment we make the peer review history of every article we publish publicly available.

When an article is published we post the peer reviewers' comments and the authors' responses online. We also post the versions of the paper that were used during peer review. These are the versions that the peer review comments apply to.

The versions of the paper that follow are the versions that were submitted during the peer review process. They are not the versions of record or the final published versions. They should not be cited or distributed as the published version of this manuscript.

BMJ Open is an open access journal and the full, final, typeset and author-corrected version of record of the manuscript is available on our site with no access controls, subscription charges or pay-per-view fees (http://bmjopen.bmj.com).

If you have any questions on BMJ Open's open peer review process please email info.bmjopen@bmj.com

BMJ Open

The ImmunoSep (Personalized Immunotherapy in Sepsis) international double-blind, double-dummy, placebo-controlled randomized clinical trial: study protocol

Journal:	*BMJ Open*
Manuscript ID	bmjopen-2022-067251
Article Type:	Protocol
Date Submitted by the Author:	12-Aug-2022

Complete List of Authors:	Kotsaki, Antigone; National and Kapodistrian University of Athens, 4th Department of Internal Medicine<br>Pickkers, Peter; Radboudumc, Intensive Care Medicine<br>Bauer, Michael; Universitätsklinikum Jena, Klinik für Anästhesiologie und Intensivtherapie<br>Calandra, Thierry; CHUV<br>Lupse, Mihaela; University of Cluj-Napoca, Department of Internal Medicine<br>Wiersinga, Joost; Amsterdam University Medical Centres, Department of Internal Medicine<br>Meylan, Sylvain; CHUV, Internal Medicine<br>Bloos, Frank; Friedrich-Schiller-Universitat Jena, Klink fur Anaesthesiologie und Intensivtherapie<br>van der Poll, Tom; Amsterdam University Medical Centres, The Center of Experimental and Molecular Medicine<br>Slim, Marleen; Amsterdam UMC Locatie AMC, Intensive Care Unit<br>van Mourik, Niels; Amsterdam UMC Location AMC, Intensive Care<br>Müller, Marcella; Amsterdam UMC Location AMC, Intensive Care<br>van Vught, Lonneke; Amsterdam University Medical Center, Department of Infectious Diseases<br>Vlaar, Alexander; Amsterdam UMC Locatie AMC, Intensive Care<br>de Nooijer, Aline; Radboudumc<br>Bakkerus, Lieke; Radboud University Medical Center, Department of Internal Medicine and Infectious Diseases<br>Weis, Sebastian; Jena University Hospital, Center for Infectious Disease and Infection Control<br>Antonakos, Nikolaos; National and Kapodistrian University of Athens, 4th Department of Internal Medicine<br>Netea, M; Radboud University Medical Center<br>Giamarellos-Bourboulis, Evangelos; National and Kapodistrian University of Athens, 4th Department of Internal Medicine

Keywords:	Adult intensive & critical care < ANAESTHETICS, INFECTIOUS DISEASES, IMMUNOLOGY, Adult intensive & critical care < INTENSIVE & CRITICAL CARE

SCHOLARONE™
Manuscripts

The ImmunoSep (Personalized Immunotherapy in Sepsis) international double-blind, double-dummy, placebo-controlled randomized clinical trial: study protocol

Antigone Kotsaki[1,2], Peter Pickkers[3], Michael Bauer[4], Thierry Calandra[5], Mihaela Lupse[6], W. Joost Wiersinga[7], Sylvain Meylan[5], Frank Bloos[4], Tom van der Poll[7], Marleen Slim[7], Niels van Mourik[7], Marcella C. A. Müller[8], Lonneke van Vught[7], Alexander P. J. Vlaar[8], Aline de Nooijer[9], Lieke Bakkerus[9], Sebastian Weis[4], Nikolaos Antonakos[1,2], Mihai G. Netea[9,10]*, Evangelos J. Giamarellos-Bourboulis[1,2]*

[1]4th Department of Internal Medicine, National and Kapodistrian University of Athens, Medical School, Greece;

[2]Hellenic Institute for the Study of Sepsis, Greece;

[3]Intensive Care Unit, Radboud University Medical Center, Nijmegen, The Netherlands;

[4]Department of Anesthesiology and Intensive Care Medicine, Jena University Hospital, Friedrich-Schiller University, Jena, Germany;

[5]Department of Infectious Diseases, Centre Hospitalier Universitaire Vaudois, Lausanne, Switzerland;

[6]Department of Internal Medicine, University of Cluj-Napoca, Romania;

[7]Department of Infectious Diseases, Amsterdam University Medical Center, The Netherlands;

[8]Intensive Care Unit, Amsterdam University Medical Center, The Netherlands;

[9]Department of Internal Medicine and Infectious Diseases, Radboud University Medical Center, Nijmegen, The Netherlands; and

[10]Department of Immunology and Metabolism, Life and Medical Sciences Institute (LIMES), University of Bonn, Germany

* These two authors contributed equally

For peer review only

Correspondence

Evangelos J. Giamarellos-Bourboulis

4th Department of Internal Medicine, ATTIKON University General Hospital

1 Rimini Street 124, 62 Athens, Greece

Tel: 0030 210 58 31 994

Fax: 0030 210 53 26 446

email: egiamarel@med.uoa.gr

For peer review only

Abstract

Introduction: Sepsis is a major cause of death among hospitalized patients. Accumulating evidence suggests that immune response during sepsis cascade lies within a spectrum of dysregulated host responses. On the one side of the spectrum there are patients whose response is characterized by fulminant hyper-inflammation or macrophage activation-like syndrome (MALS), and on the other side patients whose immune response is characterized by immunoparalysis. A sizeable group of patients are situated between the two extremes. Recognizing immune endotype is very important in order to choose the appropriate immunotherapeutic approach for each patient that gives the best chance to improve the outcome.

Methods and Analysis ImmunoSep is a randomized placebo-controlled phase 2 clinical trial with a double-dummy design in which the effect of precision immunotherapy on sepsis phenotypes with MALS or immunoparalysis is studied. Patients are stratified using biomarkers. Specifically, two hundred eighty patients will be 1:1 randomly assigned to placebo or active immunotherapy as adjunct to standard-of-care treatment. In the active immunotherapy arm, patients with MALS will receive Anakinra (recombinant interleukin-1 receptor antagonist) intravenously, and patients with immunoparalysis will receive subcutaneously recombinant human interferon-gamma. The primary endpoint is the comparative decrease of the mean total SOFA (Sequential Organ Failure Assessment) score by at least 1.4 points by day 9 from randomization.

Ethics and dissemination: The protocol is approved by National Ethics Committee of Greece and by the National Organization for Medicines of Greece; by the Central Committee on Research Involving Human Subjects (CCMO) and METC Oost Netherland for the Netherlands; and by Commission Cantonale d’éthique de la

For peer review only

recherche sur l'être human (CER-VD) of Switzerland. The results will be submitted for publication in peer review journals.

Trial registration

Clinicaltrials.gov NCT04990232

Key words; immunomodulation; sepsis; macrophage activation-like syndrom; immunoparalysis; anakinra; rhIFNγ

Strengths and limitation of this study

- ImmunoSep is a randomized placebo-controlled phase 2 clinical trial with a double-dummy design in which the effect of immunotherapy tailored to specific immune endotypes is studied
- This is the first precision medicine trial in sepsis, in which the patients are stratified based on the immune endotypes in macrophage activation-like syndrome (MALS) and immunoparalysis. Patient stratification relies on specific immune biomarkers.
- The immunotherapeutic approaches rely on already available drugs with well-known safety profiles: intravenous anakinra for MALS and subcutaneous rhIFNγ for immunoparalysis
- ImmunoSep is exploring the use the decrease of mean SOFA score by day 9 as a novel endpoint for sepsis trials.
- ImmunoSep also aims for the identification of novel biomarkers through the integrative analysis of transcriptomics, proteomics and metabolomics to better classify patients and develop surrogates of treatment response.
- The main limitations are the complex process of delivery of double-dummy treatment and the demanding patients visit procedures

For peer review only

INTRODUCTION

Sepsis is a life-threatening organ dysfunction condition that results from the dysregulated host response to a severe infection[1]. Patients with sepsis-induced dysregulation present a broad spectrum of perturbation ranging from immune hyper-activation to immune suppression. In this respect, approximately 5 to 10% of patients present mainly with fulminant hyperinflammation, an entity also known as macrophage activation-like syndrome (MALS)[2], whereas a sizeable minority of other patients have mainly ineffective responses to secondary infections, a condition described as immunoparalysis[3].

There is accumulating evidence that delivery of targeted immunotherapy for patients who present with these two extremes may improve outcome. Indeed, the post-hoc analysis of a randomized clinical trial (RCT) conducted more than 25 years ago showed that treatment with anakinra, the recombinant antagonist of the interleukin-1 receptor, provided 30% decrease of 28-day mortality among patients with hepatobiliary dysfunction and disseminated intravascular coagulation who bear phenotypic characteristics compatible with MALS[4]. During the last years we have suggested that serum ferritin can be an important diagnostic tool for MALS. Studying 5,121 patients who were split into one test and into one validation cohort and studying another confirmation cohort from Sweden, it has been found that serum concentrations of ferritin greater than 4,420 ng/ml had sensitivity 97.1% and 98% negative predictive value for the classification of MALS[2].

On the other hand, the decrease of the expression of the human leukocyte antigen (HLA)-DR expression on the membrane of circulating monocytes is considered one of the hallmarks of immunoparalysis resulting in a dysfunctional immune response, which in turns leads to susceptibility for secondary infections, prolonged hospitalization

For peer review only

and increased mortality[5]. The presence of immunoparalysis in sepsis patients is associated with at least 50% risk of death in the subsequent 28 days[6]. Evidence from human volunteers subjected to an endotoxin challenge suggests that immunoparalysis can be reversed by recombinant human interferon gamma (rhIFNγ)[7]. In addition, when rhIFNγ was administered in nine patients with septic shock, reversal of immunoparalysis was also achieved[8].

Based on the existing evidence, experts from five European countries (Germany, Greece, the Netherlands, Romania and Switzerland) designed a double-blind, double-dummy RCT with the aim to deliver personalized immunotherapy as adjunctive treatment to standard-of-care. The acronym of the trial is ImmunoSep (EudraCT number: 2020-005768-74; Clinicaltrials.gov NCT04990232) and it is funded by the Horizon 2020 program of the European Union.

Objectives

ImmunoSep is a randomized placebo-controlled phase 2 clinical trial with a double-dummy design in which the effect of personalized immunotherapy in patients with sepsis and either MALS or immunoparalysis is studied. The primary hypothesis is that the efficacy of immunotherapy in sepsis depends on the specific immune endotype of each patient, and patient stratification for administration of adjunctive immunotherapy aiming to reverse MALS and sepsis-induced immunoparalysis improves chances for a better outcome when compared with a one-size-fits-all immunotherapy approach. It is anticipated that organ dysfunctions as expressed by the mean SOFA (Sequential Organ Failure Assessment) score will be improved by day 9 after randomization.

For peer review only

METHODS AND ANALYSIS

Study design and setting

ImmunoSep is a prospective randomized placebo-controlled phase 2 clinical trial in a total number of 24 study sites in Greece, Germany, Switzerland, the Netherlands and Romania aiming to assess whether personalized adjunctive immunotherapy directed against a state of either fulminant hyperinflammation or immunoparalysis is able to improve sepsis outcomes. Patients will be selected by a panel of biomarkers and laboratory findings and will be allocated to placebo or immunotherapy treatment according to their needs by 1:1 ratio. The study enrolment will be competitive between the participating study sites targeting 280 participants.

Study Population

Inclusion and Exclusion criteria determining the eligibility of study participants are reported in Box 1.

Study procedures

Patients eligible for the study are patients either admitted to hospital from the emergency department or patients already hospitalized in the general clinical wards or in intensive care units. Once a patient is presenting with at least two of the signs of the systemic inflammatory response syndrome (SIRS) or at least one point of the quick SOFA score, then he/she or a legal representative in case the patient cannot consent, are asked for written informed consent. Trial procedures and flow are summarized in Figure 1. When the patient does not meet any exclusion criteria, he/she is screened for the presence of lower respiratory tract infection (community-acquired pneumonia, hospital-acquired pneumonia of ventilator-associated pneumonia) or primary

For peer review only

Box 1 Inclusion and exclusion criteria of the ImmunoSep trial

Inclusion criteria (patients should meet ALL of them)

- Age equal to or above 18 years.
- Both genders.
- In case of women, unwillingness to become pregnant during the study period; birth control measures apply.
- Written informed consent provided by the patient or by one first-degree relative/spouse in case of patients unable to consent.
- Community-acquired pneumonia (CAP) or hospital-acquired pneumonia (HAP) or ventilator-associated pneumonia (VAP) or primary bacteremia (BSI).
- Sepsis defined by the Sepsis-3 definitions.
- Patients with signs of MALS or sepsis-associated immunoparalysis
- Time from classification into sepsis by the Sepsis-3 definitions and start of blind intervention less than 72 hours.

Exclusion criteria (Patients meeting ANY of the following criteria CANNOT be enrolled)

- Age below 18 years.
- Refusal of written informed consent.
- Acute pyelonephritis or intraabdominal infection, meningitis or skin infection.
- Any stage IV malignancy.
- Neutropenia defined as an absolute neutrophil count lower than 1,500/mm^3.
- Any ‘do not resuscitate’ decision in the hospital.
- In the case of BSI, patients with blood cultures growing coagulase-negative staphylococci or skin commensals or catheter-related infections cannot be enrolled.
- Active tuberculosis (TB) as defined by the co-administration of drugs for the treatment of TB.
- Infection by the human immunodeficiency virus (HIV).
- Any primary immunodeficiency.
- Oral or intravenous intake of corticosteroids at a daily dose equal or greater than 0.4 mg/kg prednisone or greater the last 15 days.
- Any anti-cytokine biological treatment the last one month.
- Medical history of systemic lupus erythematosus.
- Medical history of multiple sclerosis or any other demyelinating disorder.
- Pregnancy or lactation. Women of child-bearing potential will be screened by a urine pregnancy test before inclusion in the study.

bacteremia[10] and for the Sepsis-3 definition[11]. If the patient meets all these inclusion criteria, the patient is then screened for MALS or immunoparalysis. For this, whole blood is drawn for the measurement of ferritin by an enzyme immunosorbent assay, and for the expression of HLA-DR molecules on CD45/CD14-monocytes using the Quantibrite assay by flow cytometry. Screened patients can be classified into three immune endotypes: (a) MALS defined as serum ferritin >4,420 ng/ml; (b) Immunoparalysis with serum ferritin 4,420 ng/ml or lower and less than 5,000 HLA-DR molecules on circulating CD45/CD14-monocyte; (c) unclassified. If a patient meets the criteria for both MALS and immunoparalysis, MALS is considered as the dominant diagnosis due to the higher mortality in this condition. Patients of immunogroups (a) and (b) may be enrolled in the trial provided they meet the time difference of less than 72 hours from onset of sepsis.

Study Intervention/Allocation to blind treatment

Once a patient is considered eligible for enrolment, he will be allocated blindly 1:1 to one of the two groups of treatment. Randomization is stratified by study site. The preparation of the study drug will be done in each study site by the un-blinded pharmacist investigators. The two groups of treatment are:

- **Placebo.** In addition to standard-of-care treatment decided by the attending physicians, patients will also receive intravenously (IV) 20ml (10ml for patients when creatinine clearance is lower than 30ml/min) 0.9% saline (N/S) three times daily (every eight hours) for 15 days and 0.5 ml subcutaneously (sc) 1ml 0.9% N/S every other day for a total of 15 days.
- **Active immunotherapy.** In addition to standard-of-care treatment decided by the attending physicians, patients with MALS will receive 200 mg anakinra every eight

For peer review only

hours and sc placebo as specified above whereas patients with immunoparalysis will receive IV placebo as specified above and sc 100 μg rhIFNγ once every other day for a total of 15 days. Creatinine clearance should be calculated daily by the Cockcroft Gault equation and when it is lower than 30 ml/min, anakinra will be given at half dose.

Masking protocol

The preparation of the study drug will be done in each study site by the un-blinded pharmacist investigators. Syringes with active drug or placebo will be covered to conceal the identity of the test article. The un-blinded pharmacist will provide the covered syringes to the blinded nurse or blinded investigator who will administer the infusion

Study Procedures

An overview of all study procedures is provided in Table 1. Briefly visits 1-15, visit 21, visit 28 and visit 90 include recording of co-administered drugs; SOFA score; vital signs; absolute blood cell count and differentiation, hemoglobin and absolute platelet count (if available); biochemistry (if available); coagulation (if available); blood gasses (if available), microbiology and antibiogram (if available). Recording also of the clinical state of the infection making the patient eligible for the study. Of utmost importance is recording and subsequently properly reporting any adverse event/serious adverse event occurring during trial participation. At visits days 1-2-4-7-15-21-28-90 blood sampling for trial related purposes is drawn (transcriptomic, flow cytometry, cytokine production, ATAC-epigenome sequencing, proteomic analysis) and microbiome samples from skin, nares, oral cavity and rectum.

For peer review only

Table 1 Study visits

	Study visits																		
**Day**	**0**	**1**	**2**	**3**	**4**	**5**	**6**	**7**	**8**	**9**	**10**	**11**	**12**	**13**	**14**	**15**	**21**	**28**	**90**
**Obtain ICF**	X																		
**Study drug**		X	X	X	X	X	X	X	X	X	X	X	X	X	X	X			
**SOFA score**		X	X	X	X	X	X	X	X	X	X	X	X	X	X	X	X		
**Medical history**		X																	
**Clinical state of infection**							X	X	X	X	X	X	X	X	X	X	X		
**Survival**																		X	X
**Vital sings**		X	X	X	X	X	X	X	X	X	X	X	X	X	X	X	X		
**Lab tests**		X	X	X	X	X	X	X	X	X	X	X	X	X	X	X	X		
**Microbiology**		X	X	X	X	X	X	X	X	X	X	X	X	X	X	X	X		
**Blood collection**		X	X		X			X								X	X	X	X
**Microbiome samples**		X	X		X			X								X	X	X	X
**Co-administered medication**		X	X	X	X	X	X	X	X	X	X	X	X	X	X	X	X		
**Adverse events**			X	X	X	X	X	X	X	X	X	X	X	X	X	X	X	X	X

For peer review only

Study endpoints

The primary efficacy endpoint is the comparative difference in the mean total SOFA (Sequential Organ Failure Assessment) score until day 9 after randomization between the two groups of treatment.

The secondary study endpoints is the comparison of the two groups of treatment on: a) 28-day all-cause mortality; b) 90-day all-cause mortality; c) the mean total SOFA score on day 15 from randomization; d) the impact of personalized immunotherapy on the reversal of MALS or immunoparalysis on day 15 from randomization, defined for patients with MALS as at least 15% decrease of the baseline serum ferritin, and for patients with immunoparalysis as restoration of HLA-DR expression on CD45/CD14-monocytes above 8,000/cell.

Exploratory study endpoints are: a) the impact of personalized immunotherapy on the resolution of infection leading to study enrolment on day 15 after randomization; and b) the development of genomic, epigenomic, proteomic, metabolomic and microbiomic surrogate biomarkers for the primary and secondary endpoints. This will come from the exploitation of the genomic and proteomic material that will be analyzed by the partners of the ImmunoSep project.

Sample size

The study is powered for the primary endpoint, i.e. decrease of mean SOFA score by at least 1.4 points on day 9. In order to calculate the power of the study the following hypotheses are made: according to data from the previous randomized clinical trials in sepsis VISEP[9] and MAXSEP[10] on a total of 1,137 patients, there is a significant association between the mean total SOFA score at day 9 and 28-day mortality. A reduction of the primary endpoint by 1.4 points is expected to be associated

For peer review only

with a reduction of 28-day mortality. Based on the preliminary results of the PROVIDE study (Clinicaltrials.gov NCT 0333225) 40% of patients of the enrolled patients in each arm will be recruited with fulminant hyper-inflammation and another 60% for sepsis-associated immunoparalysis. The study is powered for 90% at the 5% level of significance and the anticipated mean difference in the standard deviation between the two groups will be 3.2. In order to detect this difference of 1.4 points in the mean SOFA score, 117 patients will be needed per trial arm. Considering a drop-out rate of about 15%, a total of 280 patients needs to be randomized.

Ethics and dissemination

This clinical study falls under Directive 2001/20/EC (Clinical Trials Directive). The protocol was submitted and approved by National Ethics Committee of Greece (approval 2/21); by the National Organization for Medicines of Greece (approval IS008/21); by the Central Committee on Research Involving Human Subjects (CCMO) for the Netherlands (approval NL76706.091.21); and by Commission Cantonale d'éthique de la recherche sur l'être human (CER-VD) of Switzerland (approval 2022-00606); by the German Federal Institute for Drugs and Medical Devices (BfArM, approval 2022-05-25) and by the Ethics Committee of the Jena University Hospital (approval 2022-2540-AMG-ff). The protocol is also submitted to applicable regulatory and ethics committees of Romania where approval is pending. The patients will be included after having provided a written informed consent to the investigator. If the patient is not able to understand the information given, he/she can be included if the same procedure is completed by one first degree relative/spouse /legal representative. After the patient's recovery, he/she will be asked if he/she agrees to continue the trial. Her/his consent will again be necessary for the continuation of the study. No study

For peer review only

related procedure will be performed prior to obtaining written informed consent. For the Netherlands and Germany, a separate deferred consent process for patients unable to consent is followed according to applicable legislation, as described in details in protocol supplements. The trial shall be governed by the international standards for Good Clinical Practice (GCP) developed by the International Council for Harmonization of Technical Requirements for Pharmaceuticals for Human Use, the Directive 2001/20/EC for Clinical trials and General Data Protection Regulation 679/2016 (EC).

Data collection/data management

Data will be collected on an electronic case report form (e-CRF) by a trained investigator or research assistant at each centre. Data management will be performed by the Hellenic Institute for the Study of Sepsis (HISS) according to ICHGCP, EMA/INS/GCP/454280, EMA/226170/2021 and GDPR applicable regulations. Clinical trial monitoring will be performed by clinical research associates appointed by HISS. CRAs will ensure protocol adherence, GCP compliance and maintain regular communication with both sites and sponsor during clinical trial conduct. During monitoring visits, source data verification will be carried out by CRAs and all entries in the CRFs will be compared with the original source documents ensuring data integrity.

Patient and Public Involvement

Patients or the public WERE NOT involved in the design of the ImmunoSep trial

DISCUSSION

Risk stratification and delivery of immunotherapy tailored to the needs of every patient is the backbone of the ImmunoSep RCT. ImmunoSep is already running in

For peer review only

Greece, the Netherlands and Switzerland, whereas the start in two more European countries (Germany and Romania) is pending upon final approval by the National Ethics Committees. As of June 1st 2022, 153 patients were screened and 65 patients were enrolled.

A study of precision immunotherapy tailored to the needs of every patient has two main requirements a) the mechanism driving the immune dysfunction of the host is well defined; and b) the immune state of the enrolled patients is driven by an immune endotype that can be identified by readily available biomarkers. This is the reason why only sepsis who are suffering by well-defined MALS or immunoparalysis are randomized to receive immunotherapy in the ImmunoSep trial, whereas unclassified patients that do not fulfil the immunological criteria are not enrolled.

There are major challenges in running the ImmunoSep trial which may be summarized as follows: a) the anticipated screening failure rate; and b) the utility of the primary endpoint. The two biomarkers used, ferritin and the absolute count of HLA-DR molecules on CD45/CD14-monocytes, have been used for immune classification in the previously run trial PROVIDE (Clinicaltrial.gov NCT03332225). Published results in study participants with community-acquired pneumonia validated the ability of the biomarkers to classify patients into MALS, immunoparalysis or unclassified[11]. With the use of these biomarkers, the anticipated screening failure rate based on PROVIDE study and the initial months of recruitment in ImmunoSep is anticipated to amount between 30 to 60%. Sepsis organ dysfunction is measured through the SOFA score[1]. As such, the introduction of mean SOFA score as an endpoint is reflecting the ability of immunotherapy on restoration of sepsis-induced organ dysfunction.

ImmunoSep is the first study employing patient stratification and precision medicine in immunotherapy for sepsis, and it is anticipated that such an approach has

For peer review only

much better chances to improve the outcome of the patients compared with earlier one-size-fits-all clinical trials. Although similar interventions of precision medicine have not yet been performed and registered for sepsis, anakinra has recently been licensed by the European Medicines Agency for adults with pneumonia by the SARS-CoV-2 coronavirus. Treatment is guided by circulating concentrations of the biomarker suPAR (soluble urokinase plasminogen activator receptor) of 6 ng/ml or more, which is an indicator of the early activation of the IL-1 cascade[12]. Using such precision medicine approach, anakinra treatment was able to significantly decrease 0.36 times the risk for a worse score on the 11-point WHO-CPS at day 28 (95% confidence interval 0.26–0.50, $P < 0.0001$). compared to patients receiving placebo[13]. So, a trial using an elevated suPAR concentration as an enrolment criterium showed improved outcomes, while trials not using an enrichment strategy did not[14], plausibly reflecting the relevance of phenotyping.

Several recent data generate hope that the administration of anakinra and rhIFNγ may improve outcomes for patients with critical COVID-19. In recent open-label trial, patients with COVID-19 pneumonia classified with MALS using ferritin more than 4,420 ng/ml were treated for seven days with IV anakinra 200mg every wight hours; mortality was decreased compared to historical comparators[15]. In five COVID-19 patients with persistently low HLA-DR expression and incapacity to eliminate the virus, subcutaneous treatment with rhIFNγ led to considerable viral elimination, clinical improvement and discharge from the Intensive Care Unit[16].

ImmunoSep is a promising approach aiming to change clinical practice for the management of the critically ill patients with sepsis by using patient stratification and precision medicine. Appropriate identification of immune endotypes with biomarkers

For peer review only

and delivery of treatment tailored to patients' is likely to represent the future of adjuvant immunotherapy for sepsis and other severe infections.

FULL REFERENCES

1. Singer M, Deutschman CS, Seymour CW, et al. The Third International Consensus Definitions for Sepsis and Septic Shock (Sepsis-3). *JAMA* 2016; 315: 801-810
2. Kyriazopoulou E, Leventogiannis K, Norrby-Teglund A, et al. Macrophage activation-like syndrome: an immunological entity associated with rapid progression to death in sepsis. *BMC Med* 2017; 15: 172
3. Antonakos N, Tsaganos T, Oberle V, et al. Decreased cytokine production by mononuclear cells after severe Gram-negative infections: early clinical signs and association with final outcome. *Crit Care* 2017, 21: 48
4. Shakoory B, Carcillo JA, Chatham WW, et al. Interleukin-1 receptor blockade is associated with reduced mortality in sepsis patients with features of macrophage activation syndrome: reanalysis of a prior phase III trial. *Crit Care Med* 2016; 44: 275-281
5. Hotchkiss RS, Monneret G, Payen D. Immunosuppression in sepsis: a novel understanding of the disorder and a new therapeutic approach. *Lancet Infect Dis* 2013, 13: 260-268
6. Deutschman CS, Tracey KJ. Sepsis: current dogma and new perspectives. *Immunity* 2014; 40: 463-475

For peer review only

7. Leentjens J, Kox M, Koch RM, et al. Reversal of immunoparalysis in humans in vivo: a double-blind, placebo-controlled, randomized pilot study. *Am J Respir Crit Care Med* 2012; 186: 838-845

8. Döcke WD, Randow F, Syrbe U, et al. Monocyte deactivation in septic patients: restoration by IFN-γ treatment. *Nat Med* 1997; 3: 678-681

9. Brunkhorst FM, Engel C, Bloos F, et al. Intensive insulin therapy and pentastarch resuscitation in severe sepsis. *N Engl J Med* 2008; 358: 125-139

10. Brunkhorst FM, Oppert M, Marx G, et al. Effect of empirical treatment with moxifloxacin and meropenem vs meropenem on sepsis-related organ dysfunction in patients with severe sepsis. *JAMA* 2012; 307: 2390-2399

11. Giamarellos-Bourboulis EJ, Netea MG, Rovina N, et al. Complex immune dysregulation in COVID-19 patients with severe respiratory failure. *Cell Host Microbe* 2020, 27: 992-1000

12. Renieris G, Karakike E, Gkavogianni T, et al. IL-1 mediates tissue-specific inflammation and severe respiratory failure in COVID-19. *J Innate Immun* 2022; doi: 10.1159/000524560

13. Kyriazopoulou E, Poulakou G, Milionis H, et al. Early treatment of COVID-19 with anakinra guided by soluble urokinase plasminogen activator receptor: a double-blind, randomized controlled phase 3 trial. *Nat Med* 2021; 27: 1752-1760

14. Declercq J, Van Damme KFA, De Leeuw E, Maes B, Bosteels C, Tavernier SJ, De Buyser S, Colman R, Hites M, Verschelden G *et al*: Effect of anti-interleukin drugs in patients with COVID-19 and signs of cytokine release syndrome (COV-AID): a factorial, randomised, controlled trial**.** *Lancet Respir Med* 2021; 9 : 1427-1438

For peer review only

15. Karakike E, Dalekos GN, Koutsodimitropoulos I, et al. ESCAPE: an open-label trial of personalized immunotherapy in critically ill COVID-19 patients. *J Innate Imm* 2022; 14: 218-228

16. Van Laarhoven A, Kurver L, Overheul GJ, et al. Interferon gamma immunotherapy in five critically ill COVID-19 patients with impaired cellular immunity: A case series. *Med* 2021; 2: 1163-1170

For peer review only

Authors' contributions

AK wrote the current manuscript, edited the protocol, provided feedback for intellectual content for this manuscript and gave final approval for this manuscript to be published. PP. MB, TC, ML, WJW, SM, FB, TVDP. AV, SW, NA and MGN edited the protocol, provided feedback for intellectual content for this manuscript and gave final approval for this manuscript to be published.

EJGB wrote the protocol, provided feedback for intellectual content for this manuscript and gave final approval for this manuscript to be published.

Funding statement: This work was supported by Horizon 2020 program grant 847422

Competing interests statement

E. J. Giamarellos-Bourboulis has received honoraria from Abbott CH, bioMérieux, Brahms GmbH, GSK, InflaRx GmbH, Sobi and XBiotech Inc; independent educational grants from Abbott CH, AxisShield, bioMérieux Inc, InflaRx GmbH, Johnson & Johnson, MSD, Sobi and XBiotech Inc.; and funding from the Horizon2020 Marie-Curie Project European Sepsis Academy (granted to the National and Kapodistrian University of Athens), and the Horizon 2020 European Grants ImmunoSep and RISKinCOVID (granted to the Hellenic Institute for the Study of Sepsis). M.G Netea is a scientific founder and member of scientific advisory board of Trained Therapeutics Discovery and Lemba.

Word Count: 2,594 words

For peer review only

Figure 1: Screening process for patient eligibility for enrolment in the ImmunoSep study

For peer review only

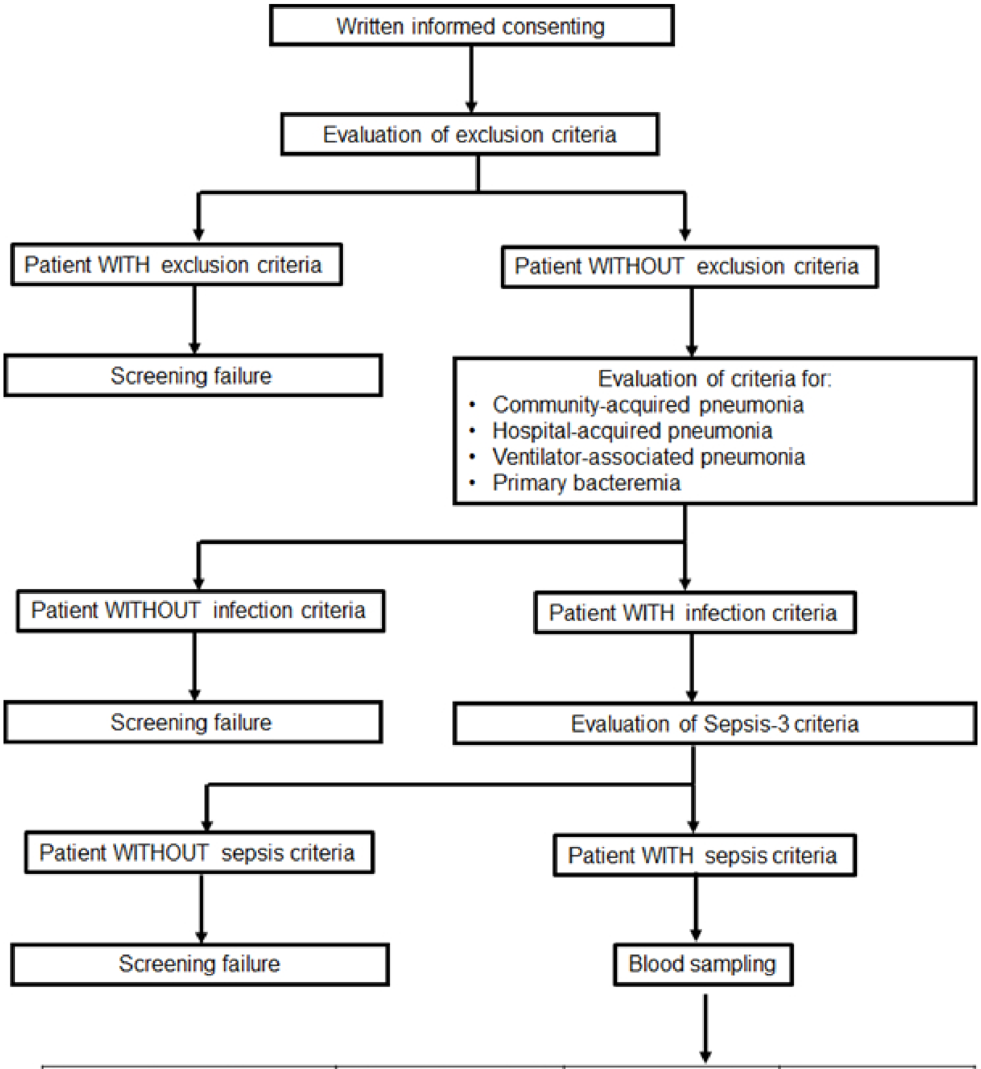

**Immune classification**	**Hyper-inflammation/ Randomize**		**Unclassified Do NOT randomize**	**Immunoparalysis/ Randomize**
Ferritin (ng/ml)	>4,420	>4,420	≤4,420	≤4,420
HLA-DR (AB per CD14-cell)	≥5,000	<5,000	≥5,000	<5,000

Screening process for patient eligibility for enrolment in the ImmunoSep study

160x197mm (96 x 96 DPI)

BMJ Open

The ImmunoSep (Personalized Immunotherapy in Sepsis) international double-blind, double-dummy, placebo-controlled randomized clinical trial: study protocol

Journal:	*BMJ Open*
Manuscript ID	bmjopen-2022-067251.R1
Article Type:	Protocol
Date Submitted by the Author:	13-Nov-2022
Complete List of Authors:	Kotsaki, Antigone; National and Kapodistrian University of Athens, 4th Department of Internal Medicine<br>Pickkers, Peter; Radboudumc, Intensive Care Medicine<br>Bauer, Michael; Universitätsklinikum Jena, Klinik für Anästhesiologie und Intensivtherapie<br>Calandra, Thierry; CHUV<br>Lupse, Mihaela; University of Cluj-Napoca, Department of Internal Medicine<br>Wiersinga, Joost; Amsterdam University Medical Centres, Department of Internal Medicine<br>Meylan, Sylvain; CHUV, Internal Medicine<br>Bloos, Frank; Friedrich-Schiller-Universitat Jena, Klink fur Anaesthesiologie und Intensivtherapie<br>van der Poll, Tom; Amsterdam University Medical Centres, The Center of Experimental and Molecular Medicine<br>Slim, Marleen; Amsterdam UMC Locatie AMC, Intensive Care Unit<br>van Mourik, Niels; Amsterdam UMC Location AMC, Intensive Care<br>Müller, Marcella; Amsterdam UMC Location AMC, Intensive Care<br>van Vught, Lonneke; Amsterdam University Medical Center, Department of Infectious Diseases<br>Vlaar, Alexander; Amsterdam UMC Locatie AMC, Intensive Care<br>de Nooijer, Aline; Radboudumc<br>Bakkerus, Lieke; Radboud University Medical Center, Department of Internal Medicine and Infectious Diseases<br>Weis, Sebastian; Jena University Hospital, Center for Infectious Disease and Infection Control<br>Antonakos, Nikolaos; National and Kapodistrian University of Athens, 4th Department of Internal Medicine<br>Netea, M; Radboud University Medical Center<br>Giamarellos-Bourboulis, Evangelos; National and Kapodistrian University of Athens, 4th Department of Internal Medicine
**Primary Subject Heading**:	Intensive care
Secondary Subject Heading:	Immunology (including allergy), Infectious diseases
Keywords:	Adult intensive & critical care < ANAESTHETICS, INFECTIOUS DISEASES, IMMUNOLOGY, Adult intensive & critical care < INTENSIVE & CRITICAL CARE

For peer review only - http://bmjopen.bmj.com/site/about/guidelines.xhtml

SCHOLARONE™
Manuscripts

The ImmunoSep (Personalized Immunotherapy in Sepsis) international double-blind, double-dummy, placebo-controlled randomized clinical trial: study protocol

Antigone Kotsaki[1,2], Peter Pickkers[3], Michael Bauer[4], Thierry Calandra[5], Mihaela Lupse[6], W. Joost Wiersinga[7], Sylvain Meylan[5], Frank Bloos[4], Tom van der Poll[7], Marleen Slim[7], Niels van Mourik[7], Marcella C. A. Müller[8], Lonneke van Vught[7], Alexander P. J. Vlaar[8], Aline de Nooijer[9], Lieke Bakkerus[9], Sebastian Weis[4, 10, 11], Nikolaos Antonakos[1,2], Mihai G. Netea[9,10]*, Evangelos J. Giamarellos-Bourboulis[1,2]*

[1]4th Department of Internal Medicine, National and Kapodistrian University of Athens, Medical School, Greece;

[2]Hellenic Institute for the Study of Sepsis, Greece;

[3]Intensive Care Unit, Radboud University Medical Center, Nijmegen, The Netherlands;

[4]Department of Anesthesiology and Intensive Care Medicine, Jena University Hospital, Friedrich-Schiller University, Jena, Germany;

[5]Department of Infectious Diseases, Centre Hospitalier Universitaire Vaudois, Lausanne, Switzerland;

[6]Department of Internal Medicine, University of Cluj-Napoca, Romania;

[7]Department of Infectious Diseases, Amsterdam University Medical Center, The Netherlands;

[8]Intensive Care Unit, Amsterdam University Medical Center, The Netherlands;

[9]Department of Internal Medicine and Infectious Diseases, Radboud University Medical Center, Nijmegen, The Netherlands;

[10]Institute for Infectious Disease and Infection Control, Jena University Hospital, Friedrich-Schiller University Jena, Germany;

[11]Leibniz Institute for Natural Product Research and Infectio, Biology, Hans-Knöll Institute (HKI), Jena, Germany; and

For peer review only

[12]Department of Immunology and Metabolism, Life and Medical Sciences Institute (LIMES), University of Bonn, Germany

* These two authors contributed equally

Correspondence

Evangelos J. Giamarellos-Bourboulis

4th Department of Internal Medicine, ATTIKON University General Hospital

1 Rimini Street 124 62 Athens, Greece

Tel: 0030 210 58 31 994

Fax: 0030 210 53 26 446

email: egiamarel@med.uoa.gr

Name and contact information for the trial sponsor

Sponsor: Hellenic Institute for the Study of Sepsis

Contact: Evangelos J. Giamarellos-Bourboulis

President of the Board

88 Michalakopoulou Str

115 28 Athens, Greece

Tel: 0030 210 74 80 660

email: president@sepsis.gr

Role of study sponsor and funder: The Sponsor has contributed in the study design; it is responsible for the collection, management, analysis, and interpretation of data; and it is responsible for writing of clinical study report. The Sponsor is also responsible to decide with the investigators to submit the report for publication. The funder does not have any role in study design; collection, management, analysis, and interpretation of data; writing of the report; and the decision to submit the report for publication.

For peer review only

Abstract

Introduction: Sepsis is a major cause of death among hospitalized patients. Accumulating evidence suggests that immune response during sepsis cascade lies within a spectrum of dysregulated host responses. On the one side of the spectrum there are patients whose response is characterized by fulminant hyper-inflammation or macrophage activation-like syndrome (MALS), and on the other side patients whose immune response is characterized by immunoparalysis. A sizeable group of patients are situated between the two extremes. Recognizing immune endotype is very important in order to choose the appropriate immunotherapeutic approach for each patient resulting in the best chance to improve the outcome.

Methods and Analysis ImmunoSep is a randomized placebo-controlled phase 2 clinical trial with a double-dummy design in which the effect of precision immunotherapy on sepsis phenotypes with MALS and immunoparalysis is studied. Patients are stratified using biomarkers. Specifically, two hundred eighty patients will be 1:1 randomly assigned to placebo or active immunotherapy as adjunct to standard-of-care treatment. In the active immunotherapy arm, patients with MALS will receive Anakinra (recombinant interleukin-1 receptor antagonist) intravenously, and patients with immunoparalysis will receive subcutaneously recombinant human interferon-gamma. The primary endpoint is the comparative decrease of the mean total SOFA (Sequential Organ Failure Assessment) score by at least 1.4 points by day 9 from randomization.

Ethics and dissemination: The protocol is approved by: the German Federal Institute for Drugs and Medical Devices; the National Ethics Committee of Greece and by the National Organization for Medicines of Greece; the Central Committee on Research Involving Human Subjects (CCMO) and METC Oost Netherland for the Netherlands;

For peer review only

the National Agency for Medicine and Medical Products of Romania; and the Commission Cantonale d'éthique de la recherche sur l'être human (CER-VD) of Switzerland. The results will be submitted for publication in peer review journals.

Trial registration

Clinicaltrials.gov NCT04990232

Key words; immunomodulation; sepsis; macrophage activation-like syndrom; immunoparalysis; anakinra; rhIFNγ

For peer review only

Date and version identifier: 04 December 2020, 1.0

Strengths and limitations of this study

- ImmunoSep is a double-blinded randomized phase 2 clinical trial with double-dummy, placebo-controlled design
- This is a personalized medicine study to demonstrate the effects of immunotherapy tailored to specific immune endotypes
- The primary endpoint is the decrease of mean SOFA score by day 9.
- ImmunoSep also aims for the identification of novel biomarkers through the integrative analysis of -omics of patients' samples.
- This trial is not powered to demonstrate an effect on mortality.

INTRODUCTION

Sepsis is a life-threatening organ dysfunction that results from the dysregulated host response to an infection[1]. Patients with sepsis-induced dysregulation present a broad spectrum of perturbation ranging from immune hyper-activation to immune suppression. In this respect, approximately 5 to 10% of patients present mainly with fulminant hyperinflammation, an entity also known as macrophage activation-like syndrome (MALS)[2], whereas a sizeable minority of other patients have mainly ineffective responses to secondary infections, a condition described as immunoparalysis[3].

There is accumulating evidence that delivery of targeted immunotherapy for patients who present with these two extremes may improve outcome. Indeed, the post-hoc analysis of a randomized clinical trial (RCT) conducted more than 25 years ago showed that treatment with anakinra, the recombinant antagonist of the interleukin-1 receptor, provided 30% decrease of 28-day mortality among patients with hepatobiliary dysfunction and disseminated intravascular coagulation who bear phenotypic characteristics compatible with MALS[4]. During the last years we have suggested that serum ferritin can be an important diagnostic tool for MALS. Studying 5,121 patients who were split into one test and into one validation cohort and studying another confirmation cohort from Sweden, it has been found that serum concentrations of ferritin greater than 4,420 ng/ml had sensitivity 97.1% and 98% negative predictive value for the classification of MALS[2].

On the other hand, the decrease of the expression of the human leukocyte antigen (HLA)-DR expression on the membrane of circulating monocytes is considered one of the hallmarks of immunoparalysis resulting in a dysfunctional immune response, which in turns leads to susceptibility for secondary infections, prolonged hospitalization

For peer review only

and increased mortality[5]. The presence of immunoparalysis in sepsis patients is associated with at least 50% risk of death in the subsequent 28 days[6]. Evidence from human volunteers subjected to an endotoxin challenge suggests that immunoparalysis can be reversed by recombinant human interferon gamma (rhIFNγ)[7]. In addition, when rhIFNγ was administered in nine patients with septic shock, reversal of immunoparalysis was also achieved[8].

Based on the existing evidence, experts from five European countries (Germany, Greece, the Netherlands, Romania and Switzerland) designed a double-blind, double-dummy RCT with the aim to deliver personalized immunotherapy as adjunctive treatment to standard-of-care. The acronym of the trial is ImmunoSep (EudraCT number: 2020-005768-74; Clinicaltrials.gov NCT04990232) and it is funded by the Horizon 2020 program of the European Union.

For peer review only

Objectives

ImmunoSep is a randomized placebo-controlled phase 2 clinical trial with a double-dummy design in which the effect of personalized immunotherapy in patients with sepsis and either MALS or immunoparalysis is studied. The primary hypothesis is that the efficacy of immunotherapy in sepsis depends on the specific immune endotype of each patient, and patient stratification for administration of adjunctive immunotherapy aiming to reverse MALS and sepsis-induced immunoparalysis improves chances for a better outcome when compared with a one-size-fits-all immunotherapy approach. It is anticipated that organ dysfunctions as expressed by the mean SOFA (Sequential Organ Failure Assessment) score will be improved by day 9 after randomization.

METHODS AND ANALYSIS

Study design and setting

ImmunoSep is a prospective randomized placebo-controlled phase 2 clinical trial in a total number of 24 academic and non-academic study sites in Greece, Germany, Switzerland, the Netherlands and Romania aiming to assess whether personalized adjunctive immunotherapy directed against a state of either fulminant hyperinflammation or immunoparalysis is able to improve sepsis outcomes. Patients will be selected by a panel of biomarkers and laboratory findings and will be allocated to placebo or immunotherapy treatment according to their needs by 1:1 ratio. The study enrolment will be competitive between the participating study sites targeting 280 participants.

Study Population

Inclusion and Exclusion criteria determining the eligibility of study participants are reported in Box 1.

Study procedures

Patients eligible for the study are patients either admitted to hospital from the emergency department or patients already hospitalized in the general clinical wards or in intensive care units. Once a patient is presenting with at least two of the signs of the systemic inflammatory response syndrome (SIRS) or at least one point of the quick SOFA score, then he/she or a legal representative in case the patient cannot consent, are asked for written informed consent. Trial procedures and flow are summarized in Figure 1. When the patient does not meet any exclusion criteria, he/she is screened

For peer review only

for the presence of lower respiratory tract infection (community-acquired pneumonia, hospital-acquired pneumonia of ventilator-associated pneumonia) or primary

Box 1 Inclusion and exclusion criteria of the ImmunoSep trial

Inclusion criteria (patients should meet ALL of them)

- Age equal to or above 18 years.
- Both genders.
- In case of women, unwillingness to become pregnant during the study period; birth control measures apply.
- Written informed consent provided by the patient or by one first-degree relative/spouse in case of patients unable to consent.
- Community-acquired pneumonia (CAP) or hospital-acquired pneumonia (HAP) or ventilator-associated pneumonia (VAP) or primary bacteremia (BSI).
- Sepsis defined by the Sepsis-3 definitions.
- Patients with signs of MALS or sepsis-associated immunoparalysis
- Time from classification into sepsis by the Sepsis-3 definitions and start of blind intervention less than 72 hours.

Exclusion criteria (Patients meeting ANY of the following criteria CANNOT be enrolled)

- Age below 18 years.
- Refusal of written informed consent.
- Acute pyelonephritis or intraabdominal infection, meningitis or skin infection.
- Any stage IV malignancy.
- Neutropenia defined as an absolute neutrophil count lower than 1,500/mm^3.
- Any ‘do not resuscitate’ decision in the hospital.
- In the case of BSI, patients with blood cultures growing coagulase-negative staphylococci or skin commensals or catheter-related infections cannot be enrolled.
- Active tuberculosis (TB) as defined by the co-administration of drugs for the treatment of TB.
- Infection by the human immunodeficiency virus (HIV).
- Any primary immunodeficiency.
- Oral or intravenous intake of corticosteroids at a daily dose equal or greater than 0.4 mg/kg prednisone or greater the last 15 days.
- Any anti-cytokine biological treatment the last one month.
- Medical history of systemic lupus erythematosus.
- Medical history of multiple sclerosis or any other demyelinating disorder.
- Pregnancy or lactation. Women of child-bearing potential will be screened by a urine pregnancy test before inclusion in the study.

bacteremia and for the Sepsis-3 definition[1]. If the patient meets all these inclusion criteria, the patient is then screened for MALS and immunoparalysis. For this, whole blood is drawn for the measurement of ferritin by an enzyme immunosorbent assay, and for the expression of HLA-DR molecules on CD14+CD45+ monocytes using the Quantibrite assay by flow cytometry (BD Biosciences, New Jersey, United States). Screened patients will be classified into three groups of immune-endotypes: (a) MALS when serum ferritin is more than 4,420 ng/ml irrespective the number of HLA-DR molecules on circulating CD14+CD45+monocytes; (b) immunoparalysis when serum ferritin is 4,420 ng/ml or lower and the number of HLA-DR molecules on circulating CD14+CD45+monocyte is less than 5,000; and (c) unclassified when serum ferritin is 4,420 ng/ml or lower and the number of HLA-DR molecules on circulating CD14+CD45+monocytes is 5,000 or more. If a patient meets the criteria for both MALS and immunoparalysis, MALS is considered as the dominant diagnosis due to the higher mortality in this condition. Patients of immunogroups (a) and (b) may be enrolled in the trial provided they meet the time difference of less than 72 hours from onset of sepsis.

Study screening is facilitated by the generated web platform at the address https://sepsisonline.org. Investigators blinded to the study intervention enter the data on the inclusion and exclusion criteria in the platform and then the screened patient receives a code. Blinded investigators do not have access to the lab results of classification into immunogroups. These laboratory results are sent by an e-mail to the unblinded investigators. Once the unblinded investigators receive the e-mail, they enter the results of ferritin and HLA-DR on the platform and they receive immediate notification if the patient is enrolled or not and, in case of enrolment, of the immunogroup (MALS, sepsis-induced immunoparalysis) and if the patient is allocated to placebo or active treatment. Separate secure usernames and passwords are

For peer review only

generated for every blinded and un-blinded investigator permitting restricted access to the platform fields according to their role in the study.

Study Intervention/Allocation to blind treatment

The un-blinded pharmacists receive information on the daily preparation of the study drug by the site https://sepsisonline.org where they have to log-in using a secure username and password. Once a patient is considered eligible for enrolment, he will be allocated blindly 1:1 to one of the two groups of treatment. The randomization is stratified by study site and it is done using one computer-generated sequencing. The preparation of the study drug will be done in each study site by the un-blinded pharmacist investigators. The two groups of treatment are:

- **Placebo.** In addition to standard-of-care treatment decided by the attending physicians, patients will also receive intravenously (IV) 20ml (10ml for patients when creatinine clearance is lower than 30ml/min) 0.9% saline (N/S) three times daily (every eight hours) for 15 days and 0.5 ml subcutaneously (sc) 1ml 0.9% N/S every other day for a total of 15 days.
- **Active immunotherapy.** In addition to standard-of-care treatment decided by the attending physicians, patients with MALS will receive 200 mg anakinra every eight hours and sc placebo as specified above whereas patients with immunoparalysis will receive IV placebo as specified above and sc 100 μg rhIFNγ once every other day for a total of 15 days. Creatinine clearance should be calculated daily by the Cockcroft Gault equation and when it is lower than 30 ml/min, anakinra will be given at half dose.

For peer review only - http://bmjopen.bmj.com/site/about/guidelines.xhtml

Masking protocol

The preparation of the study drug will be done in each study site by the un-blinded pharmacist investigators. Syringes with active drug or placebo will be covered to conceal the identity of the test article. The un-blinded pharmacist will provide the covered syringes to the blinded nurse or blinded investigator who will administer the infusion. All other sub-investigators and the patients are blinded to the assigned intervention.

Study Procedures

An overview of all study procedures is provided in Table 1. Briefly visits 1-15, visit 21, visit 28 and visit 90 include recording of co-administered drugs; SOFA score; vital signs; absolute blood cell count and differentiation, hemoglobin and absolute platelet count (if available); biochemistry (if available); coagulation (if available); blood gasses (if available), microbiology and antibiogram (if available). Recording also of the clinical state of the infection making the patient eligible for the study. Of utmost importance is recording and subsequently properly reporting any adverse event/serious adverse event occurring during trial participation. At visits days 1-2-4-7-15-21-28-90 blood sampling for trial related purposes is drawn (transcriptomic, flow cytometry, cytokine production, ATAC-epigenome sequencing, proteomic analysis) and microbiome samples from skin, nares, oral cavity and rectum. All collected samples are pseudo-anonymized using a 9-digit code. Separate coding is done per study site.

For peer review only

Table 1 Study visits

	Study visits																		
**Day**	**0**	**1**	**2**	**3**	**4**	**5**	**6**	**7**	**8**	**9**	**10**	**11**	**12**	**13**	**14**	**15**	**21**	**28**	**90**
**Obtain ICF**	X																		
**Study drug**		X	X	X	X	X	X	X	X	X	X	X	X	X	X	X			
**SOFA score**		X	X	X	X	X	X	X	X	X	X	X	X	X	X	X	X		
**Medical history**		X																	
**Clinical state of infection**							X	X	X	X	X	X	X	X	X	X	X		
**Survival**																		X	X
**Vital sings**		X	X	X	X	X	X	X	X	X	X	X	X	X	X	X	X		
**Lab tests**		X	X	X	X	X	X	X	X	X	X	X	X	X	X	X	X		
**Microbiology**		X	X	X	X	X	X	X	X	X	X	X	X	X	X	X	X		
**Blood collection**		X	X		X			X								X	X	X	X
**Microbiome samples**		X	X		X			X								X	X	X	X
**Co-administered medication**		X	X	X	X	X	X	X	X	X	X	X	X	X	X	X	X		
**Adverse events**			X	X	X	X	X	X	X	X	X	X	X	X	X	X	X	X	X

For peer review only

For peer review only - http://bmjopen.bmj.com/site/about/guidelines.xhtml

Study endpoints

The primary efficacy endpoint is the difference in the mean total SOFA (Sequential Organ Failure Assessment) score until day 9 after randomization between the two groups of treatment. The time frame of 9 days for the assessment of the primary endpoint is based on previous experience coming from the VISEP[9] and MAXSEP[10] randomized clinical trials where this endpoint was used to assess an impact of treatment on sepsis-induced organ dysfunction at day 9.

The secondary study endpoints is the comparison of the two groups of treatment on: a) 28-day all-cause mortality; b) 90-day all-cause mortality; c) the mean total SOFA score on day 15 from randomization; d) the impact of personalized immunotherapy on the reversal of MALS or immunoparalysis on day 15 from randomization, defined for patients with MALS as at least 15% decrease of the baseline serum ferritin, and for patients with immunoparalysis as restoration of HLA-DR expression on CD45/CD14-monocytes above 8,000/cell. The assessment of the mean total SOFA score on day 15 from randomization is a read-out of treatment efficacy at the end of treatment. It is considered that a later timepoint of assessment of the mean total SOFA score is not needed since mortality is the secondary endpoint already assessed at a later timepoint.

Exploratory study endpoints are: a) the impact of personalized immunotherapy on the resolution of infection leading to study enrolment on day 15 after randomization; and b) the development of genomic, epigenomic, proteomic, metabolomic and microbiomic surrogate biomarkers for the primary and secondary endpoints. This will come from the exploitation of the genomic and proteomic material that will be analyzed by the partners of the ImmunoSep project.

For peer review only

Sample size

The study is powered for the primary endpoint, i.e. decrease of mean SOFA score by at least 1.4 points on day 9. In order to calculate the power of the study the following hypotheses are made: according to data from the previous randomized clinical trials in sepsis VISEP[9] and MAXSEP[10] on a total of 1,137 patients, there is a significant association between the mean total SOFA score at day 9 and 28-day mortality. A reduction of the primary endpoint by 1.4 points is expected to be associated with a reduction of 28-day mortality. Based on the preliminary results of the PROVIDE study (Clinicaltrials.gov NCT 0333225) 40% of patients of the enrolled patients in each arm will be recruited with fulminant hyper-inflammation and another 60% for sepsis-associated immunoparalysis. The study is powered for 90% at the 5% level of significance and the anticipated mean difference in the standard deviation between the two groups will be 3.2. In order to detect this difference of 1.4 points in the mean SOFA score, 117 patients will be needed per trial arm. Considering a drop-out rate of about 15%, a total of 280 patients needs to be randomized.

Statistical analysis

The endpoints of the change of the mean SOFA score will be compared between the two groups of treatment using the Welchs' t-test for mean differences. The endpoints of time to an event will be analyzed using Cox regression analysis. Analysis will be done in the intention-to-treat population with sensitivity analysis for the per-protocol population. Missing values will be imputed by last-observation carried forward.

For peer review only

Ethics and dissemination

This clinical study falls under Directive 2001/20/EC (Clinical Trials Directive). The protocol was submitted and approved by National Ethics Committee of Greece (approval 2/21); by the National Organization for Medicines of Greece (approval IS008/21); by the Central Committee on Research Involving Human Subjects (CCMO) for the Netherlands (approval NL76706.091.21); and by Commission Cantonale d'éthique de la recherche sur l'être human (CER-VD) of Switzerland (approval 2022-00606); by the German Federal Institute for Drugs and Medical Devices (BfArM, approval 2022-05-25) and by the Ethics Committee of the Jena University Hospital (approval 2022-2540-AMG-ff); and by the National Agency for Medicine and Medical Products of Romania (approval 129E/29-09-2022). The patients will be included after having provided a written informed consent to the investigator. If the patient is not able to understand the information given, he/she can be included if the same procedure is completed by one first degree relative/spouse /legal representative. After the patient's recovery, he/she will be asked if he/she agrees to continue the trial. Her/his consent will again be necessary for the continuation of the study. No study related procedure will be performed prior to obtaining written informed consent. For the Netherlands and Germany, a separate deferred consent process for patients unable to consent is followed according to applicable legislation, as described in details in protocol supplements. The trial shall be governed by the international standards for Good Clinical Practice (GCP) developed by the International Council for Harmonization of Technical Requirements for Pharmaceuticals for Human Use, the Directive 2001/20/EC for Clinical trials and General Data Protection Regulation 679/2016 (EC). One insurance contract is already active to cover financially any harm which may be caused to an individual as a result of participation in the study.

For peer review only

The authors encourage the timely publication of scientific results in peer-reviewed journals to maximize outreach to the scientific community. All publications will be available in Open Access. It is anticipated that at least 4 major publications will be generated. The first publication will cover the results of the clinical trial and the other three publications the results of the analysis of collected biomaterial. The main author list will contain the names of all investigators and sub-investigators who contributed most to the generation of the data. Their rank in the main author list will depend on the level of contribution. A separate list containing all the names of all contributing investigators and sub-investigators in all study sites will also be published in each publication. Scientific events/conferences and other networking events will provide a valuable platform for rapid dissemination of results through oral presentations, posters and personal discussions, fostering active dialogue and direct interaction with other members of the scientific community, and pave the way for future scientific collaborations. A final symposium on immunotherapy in infections will be organized to disseminate results and pave the way for a sustainable uptake of results.

Data collection/data management

Data will be collected on an electronic case report form (e-CRF) by a trained investigator or research assistant at each centre that can be found at the address https://sepsisonline.org. Data management will be performed by the Hellenic Institute for the Study of Sepsis (HISS) according to ICHGCP, EMA/INS/GCP/454280, EMA/226170/2021 and GDPR applicable regulations. Clinical trial monitoring will be performed by clinical research associates (CRAs) appointed by HISS. CRAs will ensure protocol adherence, GCP compliance and maintain regular communication with

For peer review only

both sites and sponsor during clinical trial conduct. During monitoring visits, source data verification will be carried out by CRAs and all entries in the CRFs will be compared with the original source documents ensuring data integrity. Separate blind and unblind CRAs will be appointed for the actions of the blinded and un-blinded investigators respectively. HISS is also responsible for the pharmacovigilance of the study and for the reporting of any severe and non-severe treatment-emergent adverse events (TEAEs), as well as for the reporting of any serious unexpected adverse events (SUSARs). All SUSAR are immediately reported to all study sites and to the Ethics committees of all involved hospitals and to the committees which approved the study. An annual report of all TEAEs and SUAR is also provided to all study sites and to the Ethics committees of all involved hospitals and to the committees which approved the study. HISS will also organize audits to the top recruiting study sites by an independent third body.

One Data Safety Monitoring Board (DSMB) is active for the ImmunoSep trial since January 2022. This is composed by Professors Djillali Annane, Antonio Artigas and Adam Linder. The DSMB is planned to monitor the overall safety profile of the study when the follow-up of the first 140 patients will finish. The DSMB will decide on study continuation. Emergency unblinding for safety purposes is allowed after detailed explanation by the principal investigator.

HISS will have access to the final dataset. Access to the dataset is allowed only after contractual agreement.

Patient and Public Involvement

Patients or the public WERE NOT involved in the design of the ImmunoSep trial

For peer review only

DISCUSSION

Risk stratification and delivery of immunotherapy tailored to the needs of every patient is the backbone of the ImmunoSep RCT. ImmunoSep is already running in Gemrnay, Greece, the Netherlands, Romania and Switzerland. As of June 1st 2022, 153 patients were screened and 65 patients were enrolled.

A study of precision immunotherapy tailored to the needs of every patient has two main requirements a) the mechanism driving the immune dysfunction of the host is well defined; and b) the immune state of the enrolled patients is driven by an immune endotype that can be identified by readily available biomarkers. This is the reason why only sepsis who are suffering by well-defined MALS or immunoparalysis are randomized to receive immunotherapy in the ImmunoSep trial, whereas unclassified patients that do not fulfil the immunological criteria are not enrolled.

There are major challenges in running the ImmunoSep trial which may be summarized as follows: a) the anticipated screening failure rate; and b) the utility of the primary endpoint. The two biomarkers used, ferritin and the absolute count of HLA-DR molecules on CD45/CD14-monocytes, have been used for immune classification in the previously run trial PROVIDE (Clinicaltrial.gov NCT03332225). Published results in study participants with community-acquired pneumonia validated the ability of the biomarkers to classify patients into MALS, immunoparalysis or unclassified[11]. With the use of these biomarkers, the anticipated screening failure rate based on PROVIDE study and the initial months of recruitment in ImmunoSep is anticipated to amount between 30 to 60%. Sepsis organ dysfunction is measured through the SOFA score[1]. As such, the introduction of mean SOFA score as an endpoint is reflecting the ability of immunotherapy on restoration of sepsis-induced organ dysfunction.

For peer review only

ImmunoSep is the first study employing patient stratification and precision medicine in immunotherapy for sepsis, and it is anticipated that such an approach has much better chances to improve the outcome of the patients compared with earlier one-size-fits-all clinical trials. Although similar interventions of precision medicine have not yet been performed and registered for sepsis, anakinra has recently been licensed by the European Medicines Agency for adults with pneumonia by the SARS-CoV-2 coronavirus. Treatment is guided by circulating concentrations of the biomarker suPAR (soluble urokinase plasminogen activator receptor) of 6 ng/ml or more, which is an indicator of the early activation of the IL-1 cascade[12]. Using such precision medicine approach, anakinra treatment was able to significantly decrease 0.36 times the risk for a worse score on the 11-point WHO-CPS at day 28 (95% confidence interval 0.26–0.50, $P < 0.0001$). compared to patients receiving placebo[13]. So, a trial using an elevated suPAR concentration as an enrolment criterium showed improved outcomes, while trials not using an enrichment strategy did not[14], plausibly reflecting the relevance of phenotyping.

Several recent data generate hope that the administration of anakinra and rhIFNγ may improve outcomes for patients with critical COVID-19. In a recent open-label trial, patients with COVID-19 pneumonia classified with MALS using ferritin more than 4,420 ng/ml were treated for seven days with IV anakinra 200mg every eight hours; mortality was decreased compared to historical comparators[15]. In five COVID-19 patients with persistently low HLA-DR expression and incapacity to eliminate the virus, subcutaneous treatment with rhIFNγ led to considerable viral elimination, clinical improvement and discharge from the Intensive Care Unit[16].

ImmunoSep is a promising approach aiming to change clinical practice for the management of the critically ill patients with sepsis by using patient stratification and

For peer review only

precision medicine. Appropriate identification of immune endotypes with biomarkers and delivery of treatment tailored to patients' is likely to represent the future of adjuvant immunotherapy for sepsis and other severe infections.

Authors' contributions

AK wrote the current manuscript, edited the protocol, provided feedback for intellectual content for this manuscript and gave final approval for this manuscript to be published. PP, MB, TC, ML, WJW, SM, FB, TVDP, MS, NVM, MCAM, LVV, APJV, AND, LB, SW, NA and MGN edited the protocol, provided feedback for intellectual content for this manuscript and gave final approval for this manuscript to be published.

EJGB wrote the protocol, provided feedback for intellectual content for this manuscript and gave final approval for this manuscript to be published.

Funding statement: This work was supported by Horizon 2020 program grant 847422

Competing interests statement

E. J. Giamarellos-Bourboulis has received honoraria from Abbott CH, bioMérieux, Brahms GmbH, GSK, InflaRx GmbH, Sobi and XBiotech Inc; independent educational grants from Abbott CH, AxisShield, bioMérieux Inc, InflaRx GmbH, Johnson & Johnson, MSD, Sobi and XBiotech Inc.; and funding from the Horizon2020 Marie-Curie Project European Sepsis Academy (granted to the National and Kapodistrian University of Athens), and the Horizon 2020 European Grants ImmunoSep and RISKinCOVID (granted to the Hellenic Institute for the Study of Sepsis). M.G Netea is a scientific founder and member of scientific advisory board of Trained Therapeutics Discovery and Lemba.

Word Count: 3,457 words

For peer review only

FULL REFERENCES

1. Singer M, Deutschman CS, Seymour CW, et al. The Third International Consensus Definitions for Sepsis and Septic Shock (Sepsis-3). *JAMA* 2016; 315: 801-810
2. Kyriazopoulou E, Leventogiannis K, Norrby-Teglund A, et al. Macrophage activation-like syndrome: an immunological entity associated with rapid progression to death in sepsis. *BMC Med* 2017; 15: 172
3. Antonakos N, Tsaganos T, Oberle V, et al. Decreased cytokine production by mononuclear cells after severe Gram-negative infections: early clinical signs and association with final outcome. *Crit Care* 2017, 21: 48
4. Shakoory B, Carcillo JA, Chatham WW, et al. Interleukin-1 receptor blockade is associated with reduced mortality in sepsis patients with features of macrophage activation syndrome: reanalysis of a prior phase III trial. *Crit Care Med* 2016; 44: 275-281
5. Hotchkiss RS, Monneret G, Payen D. Immunosuppression in sepsis: a novel understanding of the disorder and a new therapeutic approach. *Lancet Infect Dis* 2013, 13: 260-268
6. Deutschman CS, Tracey KJ. Sepsis: current dogma and new perspectives. *Immunity* 2014; 40: 463-475
7. Leentjens J, Kox M, Koch RM, et al. Reversal of immunoparalysis in humans in vivo: a double-blind, placebo-controlled, randomized pilot study. *Am J Respir Crit Care Med* 2012; 186: 838-845
8. Döcke WD, Randow F, Syrbe U, et al. Monocyte deactivation in septic patients: restoration by IFN-γ treatment. *Nat Med* 1997; 3: 678-681
9. Brunkhorst FM, Engel C, Bloos F, et al. Intensive insulin therapy and pentastarch resuscitation in severe sepsis. *N Engl J Med* 2008; 358: 125-139

For peer review only

10. Brunkhorst FM, Oppert M, Marx G, et al. Effect of empirical treatment with moxifloxacin and meropenem vs meropenem on sepsis-related organ dysfunction in patients with severe sepsis. *JAMA* 2012; 307: 2390-2399

11. Giamarellos-Bourboulis EJ, Netea MG, Rovina N, et al. Complex immune dysregulation in COVID-19 patients with severe respiratory failure. *Cell Host Microbe* 2020, 27: 992-1000

12. Renieris G, Karakike E, Gkavogianni T, et al. IL-1 mediates tissue-specific inflammation and severe respiratory failure in COVID-19. *J Innate Immun* 2022; doi: 10.1159/000524560

13. Kyriazopoulou E, Poulakou G, Milionis H, et al. Early treatment of COVID-19 with anakinra guided by soluble urokinase plasminogen activator receptor: a double-blind, randomized controlled phase 3 trial. *Nat Med* 2021; 27: 1752-1760

14. Declercq J, Van Damme KFA, De Leeuw E, Maes B, Bosteels C, Tavernier SJ, De Buyser S, Colman R, Hites M, Verschelden G *et al*: Effect of anti-interleukin drugs in patients with COVID-19 and signs of cytokine release syndrome (COV-AID): a factorial, randomised, controlled trial**.** *Lancet Respir Med* 2021; 9 : 1427-1438

15. Karakike E, Dalekos GN, Koutsodimitropoulos I, et al. ESCAPE: an open-label trial of personalized immunotherapy in critically ill COVID-19 patients. *J Innate Imm* 2022; 14: 218-228

16. Van Laarhoven A, Kurver L, Overheul GJ, et al. Interferon gamma immunotherapy in five critically ill COVID-19 patients with impaired cellular immunity: A case series. *Med* 2021; 2: 1163-1170

For peer review only

Figure 1: Screening process for patient eligibility for enrolment in the ImmunoSep study

For peer review only

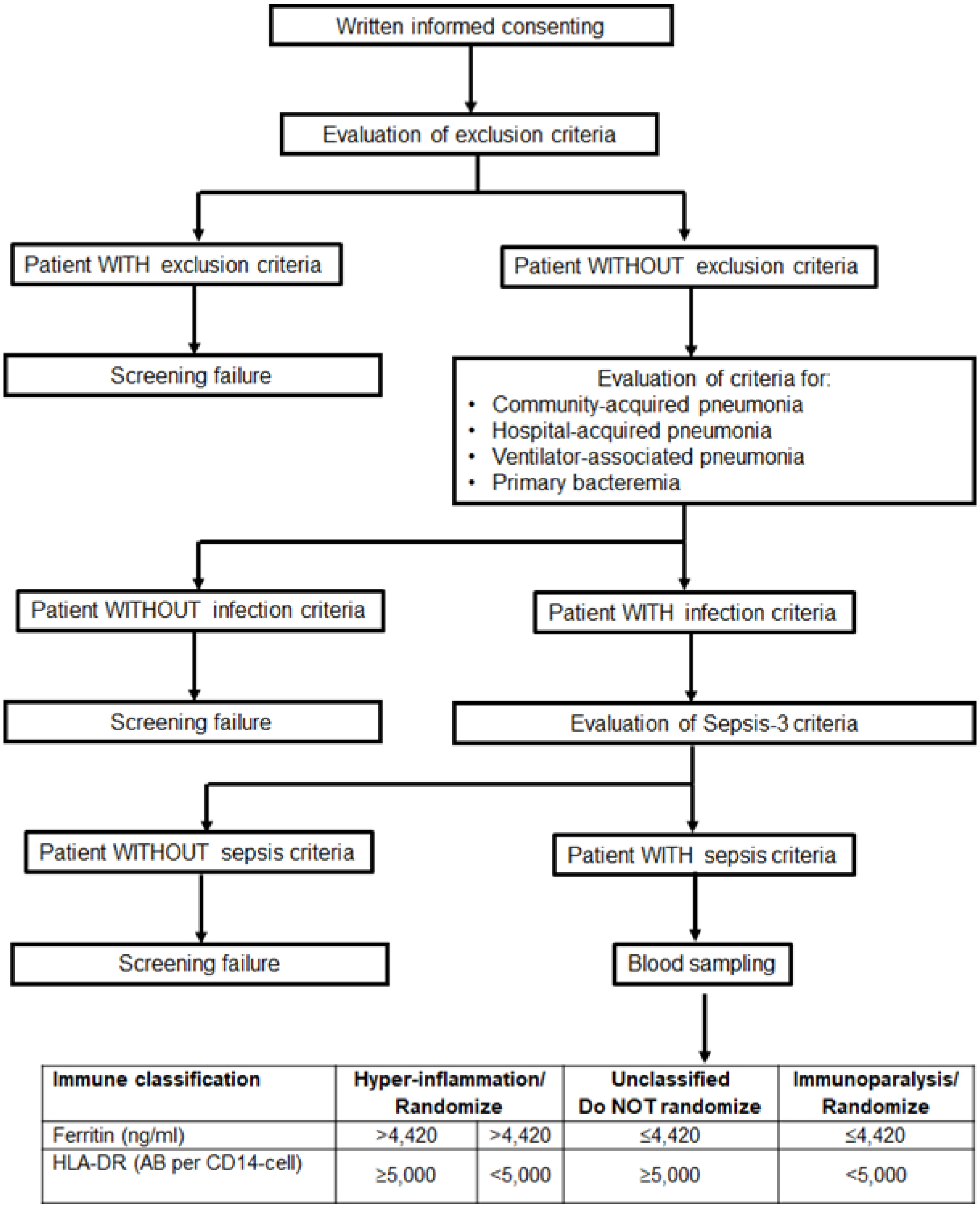

Figure 1: Screening process for patient eligibility for enrolment in the ImmunoSep study

160x197mm (96 x 96 DPI)

STANDARD PROTOCOL ITEMS: RECOMMENDATIONS FOR INTERVENTIONAL TRIALS

SPIRIT 2013 Checklist: Recommended items to address in a clinical trial protocol and related documents*

Section/item	Item No	Description	Page
**Administrative information**			
Title	1	Descriptive title identifying the study design, population, interventions, and, if applicable, trial acronym	1
Trial registration	2a	Trial identifier and registry name. If not yet registered, name of intended registry	4
	2b	All items from the World Health Organization Trial Registration Data Set	4
Protocol version	3	Date and version identifier	4
Funding	4	Sources and types of financial, material, and other support	21
Roles and responsibilities	5a	Names, affiliations, and roles of protocol contributors	1, 21
	5b	Name and contact information for the trial sponsor	3
	5c	Role of study sponsor and funders, if any, in study design; collection, management, analysis, and interpretation of data; writing of the report; and the decision to submit the report for publication, including whether they will have ultimate authority over any of these activities	2
	5d	Composition, roles, and responsibilities of the coordinating centre, steering committee, endpoint adjudication committee, data management team, and other individuals or groups overseeing the trial, if applicable (see Item 21a for data monitoring committee)	
**Introduction**			
Background and rationale	6a	Description of research question and justification for undertaking the trial, including summary of relevant studies (published and unpublished) examining benefits and harms for each intervention	5, 6
	6b	Explanation for choice of comparators	6
Objectives	7	Specific objectives or hypotheses	6

For peer review only

Trial design	8	Description of trial design including type of trial (eg, parallel group, crossover, factorial, single group), allocation ratio, and framework (eg, superiority, equivalence, noninferiority, exploratory)	6

Methods: Participants, interventions, and outcomes

Study setting	9	Description of study settings (eg, community clinic, academic hospital) and list of countries where data will be collected. Reference to where list of study sites can be obtained	7
Eligibility criteria	10	Inclusion and exclusion criteria for participants. If applicable, eligibility criteria for study centres and individuals who will perform the interventions (eg, surgeons, psychotherapists)	7-9 and Box 1
Interventions	11a	Interventions for each group with sufficient detail to allow replication, including how and when they will be administered	10
	11b	Criteria for discontinuing or modifying allocated interventions for a given trial participant (eg, drug dose change in response to harms, participant request, or improving/worsening disease)	9, 10
	11c	Strategies to improve adherence to intervention protocols, and any procedures for monitoring adherence (eg, drug tablet return, laboratory tests)	16
	11d	Relevant concomitant care and interventions that are permitted or prohibited during the trial	10
Outcomes	12	Primary, secondary, and other outcomes, including the specific measurement variable (eg, systolic blood pressure), analysis metric (eg, change from baseline, final value, time to event), method of aggregation (eg, median, proportion), and time point for each outcome. Explanation of the clinical relevance of chosen efficacy and harm outcomes is strongly recommended	13
Participant timeline	13	Time schedule of enrolment, interventions (including any run-ins and washouts), assessments, and visits for participants. A schematic diagram is highly recommended (see Figure)	11 and Figure 1
Sample size	14	Estimated number of participants needed to achieve study objectives and how it was determined, including clinical and statistical assumptions supporting any sample size calculations	14
Recruitment	15	Strategies for achieving adequate participant enrolment to reach target sample size	14

Methods: Assignment of interventions (for controlled trials)

Allocation:

For peer review only - http://bmjopen.bmj.com/site/about/guidelines.xhtml

Sequence generation	16a	Method of generating the allocation sequence (eg, computer-generated random numbers), and list of any factors for stratification. To reduce predictability of a random sequence, details of any planned restriction (eg, blocking) should be provided in a separate document that is unavailable to those who enrol participants or assign interventions	9-10
Allocation concealment mechanism	16b	Mechanism of implementing the allocation sequence (eg, central telephone; sequentially numbered, opaque, sealed envelopes), describing any steps to conceal the sequence until interventions are assigned	10
Implementation	16c	Who will generate the allocation sequence, who will enrol participants, and who will assign participants to interventions	10
Blinding (masking)	17a	Who will be blinded after assignment to interventions (eg, trial participants, care providers, outcome assessors, data analysts), and how	11
	17b	If blinded, circumstances under which unblinding is permissible, and procedure for revealing a participant's allocated intervention during the trial	17
**Methods: Data collection, management, and analysis**			
Data collection methods	18a	Plans for assessment and collection of outcome, baseline, and other trial data, including any related processes to promote data quality (eg, duplicate measurements, training of assessors) and a description of study instruments (eg, questionnaires, laboratory tests) along with their reliability and validity, if known. Reference to where data collection forms can be found, if not in the protocol	11 and Table 1
	18b	Plans to promote participant retention and complete follow-up, including list of any outcome data to be collected for participants who discontinue or deviate from intervention protocols	11 and Table 1
Data management	19	Plans for data entry, coding, security, and storage, including any related processes to promote data quality (eg, double data entry; range checks for data values). Reference to where details of data management procedures can be found, if not in the protocol	11, 16
Statistical methods	20a	Statistical methods for analysing primary and secondary outcomes. Reference to where other details of the statistical analysis plan can be found, if not in the protocol	14
	20b	Methods for any additional analyses (eg, subgroup and adjusted analyses)	14

	20c	Definition of analysis population relating to protocol non-adherence (eg, as randomised analysis), and any statistical methods to handle missing data (eg, multiple imputation)	14
**Methods: Monitoring**			
Data monitoring	21a	Composition of data monitoring committee (DMC); summary of its role and reporting structure; statement of whether it is independent from the sponsor and competing interests; and reference to where further details about its charter can be found, if not in the protocol. Alternatively, an explanation of why a DMC is not needed	17
	21b	Description of any interim analyses and stopping guidelines, including who will have access to these interim results and make the final decision to terminate the trial	15, 16
Harms	22	Plans for collecting, assessing, reporting, and managing solicited and spontaneously reported adverse events and other unintended effects of trial interventions or trial conduct	16-17
Auditing	23	Frequency and procedures for auditing trial conduct, if any, and whether the process will be independent from investigators and the sponsor	17
**Ethics and dissemination**			
Research ethics approval	24	Plans for seeking research ethics committee/institutional review board (REC/IRB) approval	15-16
Protocol amendments	25	Plans for communicating important protocol modifications (eg, changes to eligibility criteria, outcomes, analyses) to relevant parties (eg, investigators, REC/IRBs, trial participants, trial registries, journals, regulators)	15-16
Consent or assent	26a	Who will obtain informed consent or assent from potential trial participants or authorised surrogates, and how (see Item 32)	15-16
	26b	Additional consent provisions for collection and use of participant data and biological specimens in ancillary studies, if applicable	15-16
Confidentiality	27	How personal information about potential and enrolled participants will be collected, shared, and maintained in order to protect confidentiality before, during, and after the trial	15-16
Declaration of interests	28	Financial and other competing interests for principal investigators for the overall trial and each study site	23
Access to data	29	Statement of who will have access to the final trial dataset, and disclosure of contractual agreements that limit such access for investigators	17

review only

Ancillary and post-trial care	30	Provisions, if any, for ancillary and post-trial care, and for compensation to those who suffer harm from trial participation	15-16
Dissemination policy	31a	Plans for investigators and sponsor to communicate trial results to participants, healthcare professionals, the public, and other relevant groups (eg, via publication, reporting in results databases, or other data sharing arrangements), including any publication restrictions	15-16
	31b	Authorship eligibility guidelines and any intended use of professional writers	16
	31c	Plans, if any, for granting public access to the full protocol, participant-level dataset, and statistical code	16
**Appendices**			
Informed consent materials	32	Model consent form and other related documentation given to participants and authorised surrogates	Provided as Supplementary material
Biological specimens	33	Plans for collection, laboratory evaluation, and storage of biological specimens for genetic or molecular analysis in the current trial and for future use in ancillary studies, if applicable	11

*It is strongly recommended that this checklist be read in conjunction with the SPIRIT 2013 Explanation & Elaboration for important clarification on the items. Amendments to the protocol should be tracked and dated. The SPIRIT checklist is copyrighted by the SPIRIT Group under the Creative Commons "Attribution-NonCommercial-NoDerivs 3.0 Unported" license.

For peer review only
